# Supplementary material for: Dietary Antimicrobial Peptides Improve Intestinal Function, Microbial Composition and Oxidative Stress Induced by Aeromonas hydrophila in Pengze Crucian Carp (Carassius auratus var. Pengze)
Source: Antioxidants (Basel). 2022 Sep 6;11(9):1756. doi: 10.3390/antiox11091756 (PMC9495946; doi:10.3390/antiox11091756)
Supplement: Supplementary file 1 [file antioxidants-11-01756-s001.zip › antioxidants-1852232-supplementary.pdf]

Supporting appendix

Dietary antimicrobial peptides improve intestinal function, microbial composition and oxidative stress induced by *Aeromonas hydrophila* in Pengze crucian carp (*Carassius auratus* var. *Pengze*).

Shaodan Wang[1,2], Shulin Liu[1,2], Chong Wang[1,2], Bin Ye[1,2], Liqun Lv[4], Qiao Ye[5], Shaolin Xie[1,2], Guocheng Hu[3*], Jixing Zou[1,2*]

[1]Joint Laboratory of Guangdong province and Hong Kong region on Marine Bioresource Conservation and Exploitation, College of Marine Sciences, South China Agricultural University, Guangzhou 510642, China.

[2]Guangdong Laboratory for Lingnan Modern Agriculture, South China Agricultural University, Guangzhou 510642, China.

[3]State Environmental Protection Key Laboratory of Environmental Pollution Health Risk Assessment, South China Institute of Environmental Sciences, Ministry of Ecology and Environment, Guangzhou 510655, China.

[4]National Pathogen Collection Center for Aquatic Animals, Key Laboratory of Freshwater Fishery Germplasm Resources, Shanghai Ocean University, 201306 Shanghai, PR China.

[5]School of Life Sciences, Huizhou University, Huizhou 516007, Guangdong, China.

[*]Correspondence: Jixing Zou, College of Marine Sciences, South China Agricultural University, Guangzhou, 510642, China, Tel/fax: +86-20-87571321; E-mail: zoujixing@scau.edu.cn. Guocheng Hu， State Environmental Protection Key Laboratory of Environmental Pollution Health Risk Assessment, South China Institute of Environmental Sciences, Ministry of Ecology and Environment, Guangzhou 510655, China, Tel/fax: +86-20- 29119036; E-mail: huguocheng@scies.org.

Table S1. Effects of compound AMPs on intestinal morphology.

Items		G0	G1	G2	G3	G4	G5
	Villus height(μm)	628.37±12.79	638.27±24.93	618.13±48.74	584.24±29.25	620.62±11.71	599.24±5.18
	Villus width(μm)	79.09±3.98	81.94±9.47	85.75±4.29	96.47±9.65	84.32±6.95	81.72±20.00
foregut	Muscular thickness(μm)	126.77±17.89^{a}	185.42±10.65^{bc}	155.01±16.63^{ab}	175.71±21.50^{bc}	167.27±39.79^{b}	209.23±7.51^{c}
	Goblet cell number (per 100 μm)	12.50±1.67^{ab}	12.28±0.77^{ab}	13.94±3.58^{ab}	14.83±1.33^{b}	12.00±2.31^{ab}	10.61±1.70^{a}
	Villus count	27.00±1.00^{a}	30.50±0.50^{b}	25.50±0.50^{a}	27.00±1.00^{a}	27.00±1.00^{a}	30.50±0.50^{b}
	Villus height(μm)	579.52±47.31$^{\mathbf{ab}}$	555.29±19.70$^{\mathbf{ab}}$	564.18±29.35$^{\mathbf{ab}}$	528.58±3.74$^{\mathbf{a}}$	582.17±26.95$^{\mathbf{ab}}$	606.26±32.59$^{\mathbf{b}}$
	Villus width(μm)	78.84±9.47	89.19±19.21	100.35±16.62	94.51±8.19	97.94±13.48	88.57±16.19
midgut	Muscular thickness(μm)	169.15±15.66	192.76±19.34	187.55±32.46	212.80±13.78	168.60±44.90	170.14±30.87
	Goblet cell number (per 100 μm)	11.00±0.50$^{\mathbf{ab}}$	11.76±1.87$^{\mathbf{ab}}$	9.75±1.75$^{\mathbf{a}}$	13.00±0.50$^{\mathbf{b}}$	12.67±1.73$^{\mathbf{b}}$	13.58±1.58$^{\mathbf{b}}$
	Villus count	24.50±2.50	22.00±0.50	22.00±2.00	21.50±1.50	23.33±1.15	24.00±3.60
	Villus height(μm)	505.85±13.84$^{\mathbf{ab}}$	460.90±49.19$^{\mathbf{a}}$	584.02±60.32$^{\mathbf{bc}}$	568.47±0.65$^{\mathbf{bc}}$	610.47±28.44$^{\mathbf{c}}$	615.24±38.26$^{\mathbf{c}}$
	Villus width(μm)	93.87±13.21	75.22±16.88	93.42±27.38	94.92±7.50	91.25±18.64	89.81±9.88
hindgut	Muscular thickness(μm)	187.51±9.49$^{\mathbf{ab}}$	160.76±12.12$^{\mathbf{ab}}$	152.54±9.75$^{\mathbf{a}}$	294.70±33.10$^{\mathbf{c}}$	189.21±46.72$^{\mathbf{ab}}$	212.84±38.90$^{\mathbf{b}}$
	Goblet cell number (per 100 μm)	11.42±1.08	11.00±2.62	11.67±1.30	11.83±1.83	11.67±0.73	11.83±2.74
	Villus count	23.67±1.53	27.00±5.00	24.00±4.00	21.00±4.00	25.50±1.50	23.67±3.51

The values represent the means with standard errors (n=3). Values which do not have a common superscript differ significantly (P< 0.05).

Table S2. The alpha diversity index of the intestinal microbiota.

Estimators	sobs	shannon	simpson	ace	chao	coverage
G4-Mean	178.5	1.7367	0.31857	280.8	240.66	0.99783
G4-Sd	41.719	0.19241	0.054967	123.86	68.755	0.000804
G5-Mean	278	2.1248	0.24533	422.8	395.32	0.99661
G5-Sd	86.267	0.178	0.034276	55.816	120.14	0.000829
G3-Mean	272.5	2.4038	0.1927	424.87	382.33	0.99659
G3-Sd	2.1213	0.014859	0.014646	78.079	31.545	0.000402
G2-Mean	134.5	1.8656	0.26433	253.74	217.41	0.99815
G2-Sd	13.435	0.14758	0.050596	7.8811	17.987	5.02E-05
G1-Mean	229.67	2.3398	0.28857	338.25	297.81	0.99814
G1-Sd	39.374	0.811	0.20722	64.028	33.184	0.001473
G0-Mean	122.5	1.5506	0.38953	226.76	220	0.99837
G0-Sd	48.79	0.23163	0.002437	53.621	18.385	0.000552
Pvalue(G4-G5)	0.2797	0.1713	0.2509	0.2774	0.2549	0.2722
Qvalue(G4-G5)	0.2797	0.2797	0.2797	0.2797	0.2797	0.2797
Pvalue(G3-G5)	0.9364	0.1578	0.184	0.9785	0.896	0.981
Qvalue(G3-G5)	0.981	0.5519	0.5519	0.981	0.981	0.981
Pvalue(G2-G5)	0.1457	0.2538	0.7031	0.05134	0.1742	0.1191
Qvalue(G2-G5)	0.2612	0.3045	0.7031	0.2612	0.2612	0.2612
Pvalue(G1-G5)	0.4376	0.7485	0.799	0.2286	0.247	0.2852
Qvalue(G1-G5)	0.6564	0.799	0.799	0.5704	0.5704	0.5704
Pvalue(G0-G5)	0.1567	0.1087	0.02724	0.06986	0.1782	0.1298
Qvalue(G0-G5)	0.1782	0.1782	0.1634	0.1782	0.1782	0.1782
Pvalue(G3-G4)	0.08617	0.03938	0.08874	0.2986	0.1179	0.1897
Qvalue(G3-G4)	0.1768	0.1768	0.1768	0.2986	0.1768	0.2277
Pvalue(G2-G4)	0.2915	0.5304	0.4125	0.7869	0.689	0.6308
Qvalue(G2-G4)	0.7869	0.7869	0.7869	0.7869	0.7869	0.7869
Pvalue(G1-G4)	0.2573	0.3977	0.8608	0.5287	0.2837	0.8106
Qvalue(G1-G4)	0.793	0.793	0.8608	0.793	0.793	0.8608
Pvalue(G0-G4)	0.3426	0.4744	0.2097	0.6282	0.7212	0.5205
Qvalue(G0-G4)	0.7212	0.7212	0.7212	0.7212	0.7212	0.7212
Pvalue(G2-G3)	0.004822	0.03594	0.1944	0.09101	0.02339	0.03204
Qvalue(G2-G3)	0.02893	0.05392	0.1944	0.1092	0.05392	0.05392
Pvalue(G1-G3)	0.2408	0.9224	0.5792	0.2629	0.06587	0.2596
Qvalue(G1-G3)	0.3944	0.9224	0.6951	0.3944	0.3944	0.3944
Pvalue(G0-G3)	0.04913	0.03507	0.002833	0.09781	0.02437	0.06669
Qvalue(G0-G3)	0.07369	0.07014	0.017	0.09781	0.07014	0.08003
Pvalue(G1-G2)	0.05118	0.4933	0.8869	0.1759	0.05607	0.9919
Qvalue(G1-G2)	0.1682	0.7399	0.9919	0.3517	0.1682	0.9919
Pvalue(G0-G2)	0.7693	0.2462	0.073	0.5543	0.8997	0.6414
Qvalue(G0-G2)	0.8997	0.7387	0.438	0.8997	0.8997	0.8997
Pvalue(G0-G1)	0.07095	0.2906	0.56	0.1379	0.06104	0.8555
Qvalue(G0-G1)	0.2129	0.4359	0.672	0.2759	0.2129	0.8555

Table S3. Intestinal morphology after *Aeromonas hydrophila* infection.

	Items	PBS	G0	G1	G2	G3	G4	G5
foregut	Goblet cell number (per 100 μm)	11.83±2.64	14.39±1.51	15.83±0.93	14.22±1.11	14.02±3.78	13.06±1.50	13.17±3.11
midgut	Goblet cell number (per 100 μm)	12.00±0.89^{a}	15.44±2.75^{b}	14.39±1.97^{ab}	14.17±1.20^{ab}	12.22±0.98^{a}	14.06±0.96^{ab}	13.67±2.29^{ab}
hindgut	Goblet cell number (per 100 μm)	12.33±2.80^{a}	16.00±2.18^{b}	14.00±1.45^{ab}	13.28±0.79^{ab}	11.67±050^{a}	13.22±1.70^{ab}	13.00±0.60^{ab}

The values represent the means with standard errors (n=3). Values which do not have a common superscript differ significantly ($P< 0.05$).

Table S4. The goblet abundance before and after *Aeromonas hydrophila* infection.

Items	Goblet cell number (per 100 μm)					
	foregut		midgut		hindgut	
G0	12.50±1.67	14.39±1.51	11.00±0.50[a]	15.44±2.75[b]	11.42±1.08[a]	16.00±2.18[b]
G1	12.28±0.77[a]	15.83±0.93[b]	11.76±1.87	14.39±1.97	11.00±2.62	14.00±1.45
G2	13.94±3.58	14.22±1.11	9.75±1.75[a]	14.17±1.20[b]	11.67±1.30	13.28±0.79
G3	14.83±1.33	14.02±3.78	13.00±0.50	12.22±0.98	11.83±1.83	11.67±050
G4	12.00±2.31	13.06±1.50	12.67±1.73	14.06±0.96	11.67±0.73	13.22±1.70
G5	10.61±1.70	13.17±3.11	13.58±1.58	13.67±2.29	11.83±2.74	13.00±0.60

The values represent the means with standard errors (n=3). Values which do not have a common superscript differ significantly ($P< 0.05$).

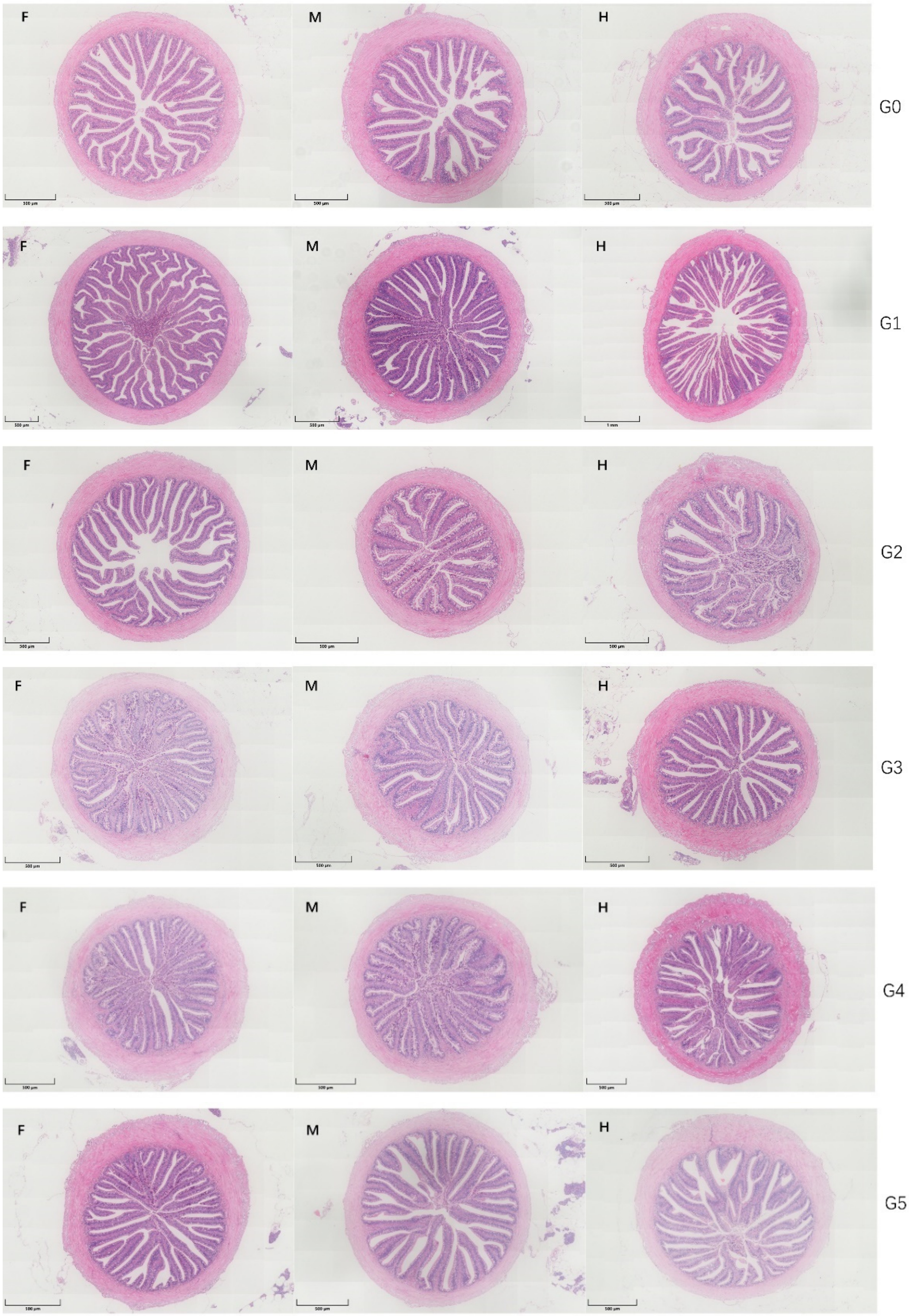

Figure S1. Morphology of intestinal of Pengze Crucian Carp between all groups. F: Foregut; M: midgut; H: hindgut. Intestinal morphology was tested by H.E. staining.

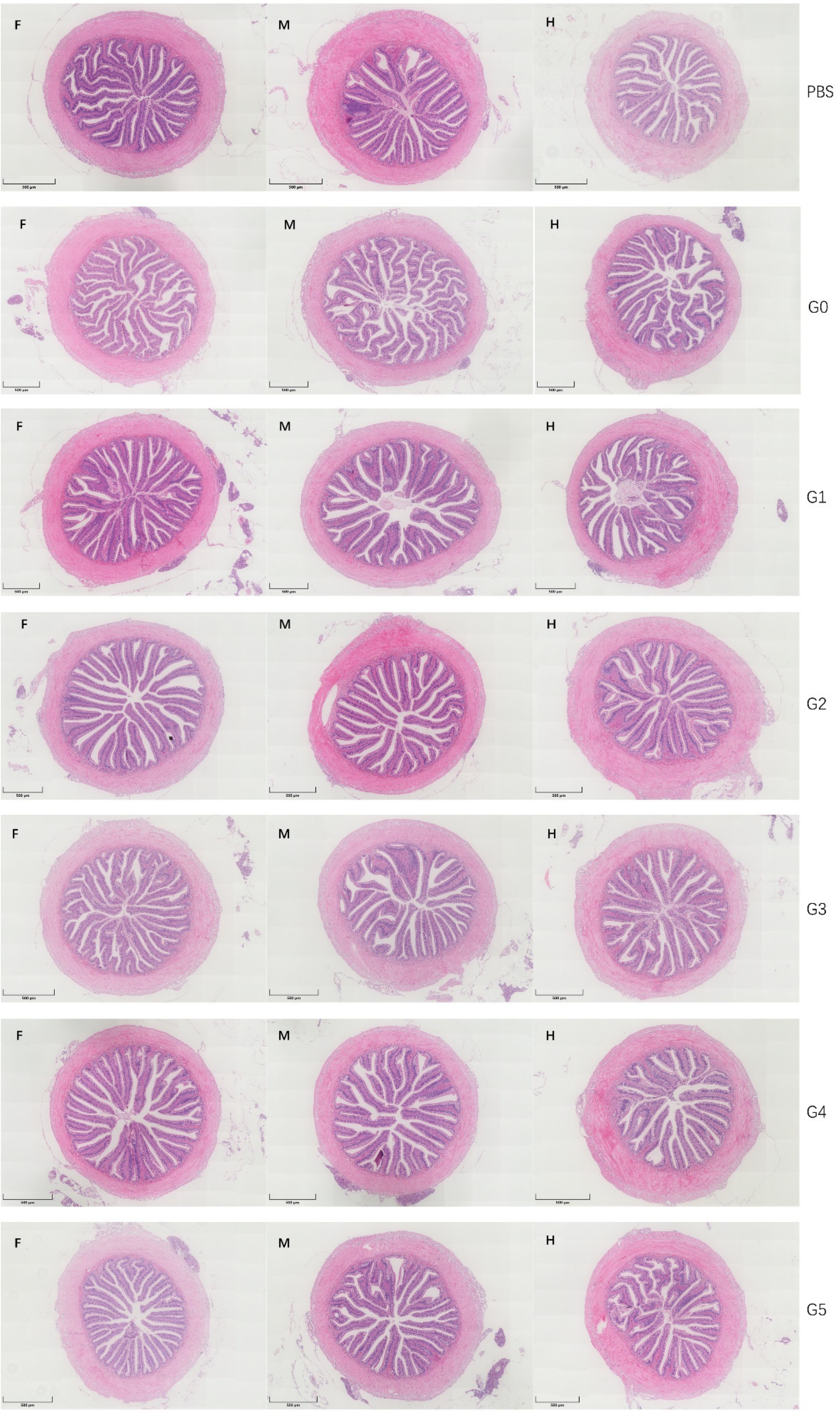

Figure S2. Intestinal morphology of each group after *Aeromonas hydrophila* infection. F: Foregut; M: midgut; H: hindgut. Intestinal morphology was tested by H.E. staining.

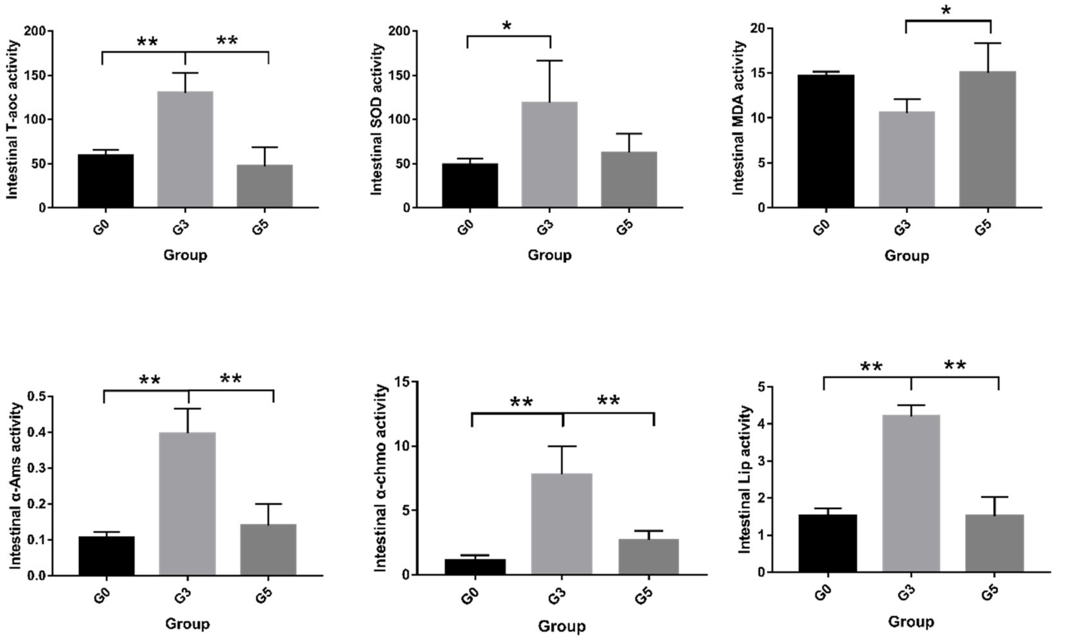

Figure S3. Comparison of Intestinal Enzyme Activities. The values represent the means with standard errors (n=3). ****P* **<0.05, *****P*** **<0.01.**

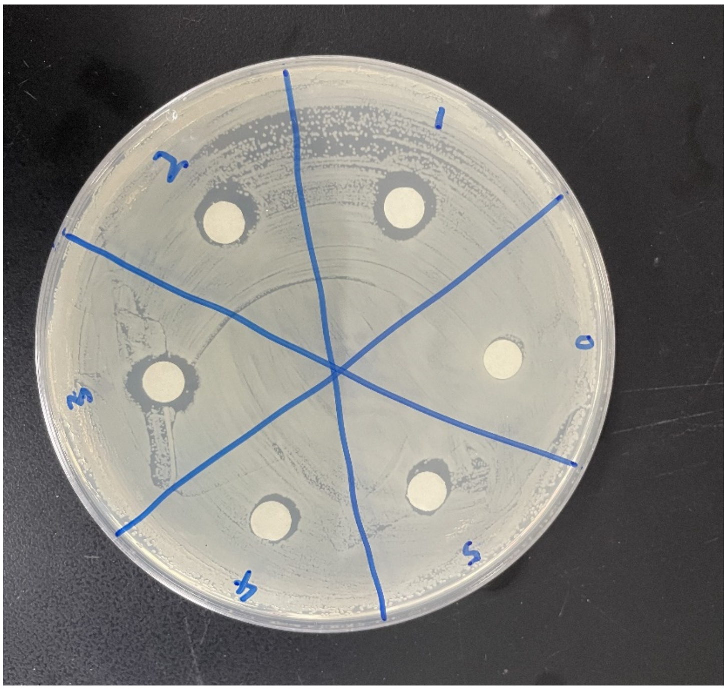

Figure S4. Antibacterial activity of *Aeromonas hydrophila* of AMPs extracts. 0: 65 % ethanol, 1: 160 mg/ml, 2: 16 mg/ml, 3: 1.6 mg/ml, 4: 0.16 mg/ml, and 5: 0.016 mg/ml.
